# A Lightweight Anonymous Client–Server Authentication Scheme for the Internet of Things Scenario: LAuth

**DOI:** 10.3390/s18113695

**Published:** 2018-10-30

**Authors:** Yuwen Chen, José-Fernán Martínez, Pedro Castillejo, Lourdes López

**Affiliations:** Departamento de Ingeniería Telemática y Electrónica (DTE), Escuela Técnica Superior de Ingeniería y, Sistemas de Telecomunicación (ETSIST), Universidad Politécnica de Madrid (UPM), C/Nikola Tesla, s/n, 28031 Madrid, Spain; jf.martinez@upm.es (J.-F.M.); pedro.castillejo@upm.es (P.C.); lourdes.lopez@upm.es (L.L.)

**Keywords:** mutual authentication, lightweight authentication, internet of things, elliptic curve cryptography, user anonymity, IoT security and privacy

## Abstract

The Internet of Things (IoT) connects different kinds of devices into a network, and enables two-way communication between devices. A large amount of data are collected by these devices and transmitted in this network, it is necessary to ensure secure communications between these devices, to make it impossible for an adversary to undermine this communication. To ensure secure communication, many authentication protocols have been proposed, in this study, a fully anonymous authentication scheme for the Internet of things scenario has been proposed, it enables the remote client to anonymously connect to the server and being serviced by the server. The proposed scheme has been verified by AVISPA and BAN Logic, and the result shows that it is safe. Besides, the simulation shows that the proposed scheme is more efficient in computation cost and communication cost.

## 1. Introduction

The Internet of Things is a network that connects all kinds of sensors, actuators, and other embedded devices. These devices can exchange data remotely via the network. A significant amount of data are collected by these devices and transmitted in this network. Among these data, there are many personal data, for example, blood pressure, pulse, and electrocardiogram, as well as home environment data, home humidity, and home temperature, etc. People are reluctant to let any party use the data without authorization. There is a need for an authentication scheme to make sure that the data is only accessible to authorized members. Authentication schemes have been studied in the past to solve this problem.

However, in some cases, mutual authentication is not sufficient for protecting the privacy of the clients. In the healthcare environment, an adversary can eavesdrop the information flow and find out which patient’s data is being transmitted. The client’s medical condition is revealed in this way. In this study, a light weighted authentication and key establishment scheme was proposed, which enables the remote client to be authenticated anonymously by the server. In the proposed scheme, we only used some light weighted security operations: XOR operations, hash functions and a minimal amount of asymmetric encryptions to fulfill perfect forward secrecy, as discussed in the previous work, these operations are relatively light weighted ones, we will continue to discuss this problem in Section 7.1. As energy consumption is of paramount importance in the context where energy are provided by small batteries, there is a high demand for a lightweight authentication scheme [1,2]. For these two reasons, we come up with this authentication scheme. Our contributions are mainly three- fold:

1. We propose a lightweight anonymous authentication for the Internet of things scenario; the scheme achieves various security features: perfect forward privacy, user anonymity, resistance to an offline dictionary attack, etc. In addition, to verify the security features of the proposed scheme, the proposed scheme is also verified by AVISPA and the BAN Logic.
2. We specially design the password changing phase, making it more efficient compared to that in the related works.
3. We simulate the proposed scheme and other related schemes using C++. The results show the communication cost and the computation cost are reduced compared with related proposals.

In Section 2, we discussed the related works, in Section 3, we introduced the proposed scheme, Section 4 and Section 5 are security analyses using AVISPA and BAN logic, Section 6 is the formal security analysis section. In Section 7, we compared the proposed scheme with related works. In Section 8, we analyzed the security features. Section 9 is the conclusion part.

## 2. Related Work

Tu et al. proposed an authentication protocol based on a smart card; the protocol is a two-factor authentication scheme based on an elliptic curve [3]. However, this scheme is found to be vulverable to impersonation attacks; an attacker can impersonate as a legal server according to Farash [4]. Ibrahim et al. proposed secure anonymous mutual authentication for star two-tier wireless body area networks [5]. Chaudhry et al. proposed a remote user authentication scheme using elliptic curve cryptography that can withstand various attacks in the internet of things scenario, for example, smart card lost attack, replay attack [6]. Kumari analyzed the scheme of Farash [7], and they found that Farash’s scheme is vulnerable to various attacks, for example, impersonation attack, password guessing attack and temporary session specific information reveal attack, etc.

Jing et al. proposed an authentication between user and server, which could protect well the identity privacy of the user [8], however, their scheme requires extra storage capacity at the server side. In the scheme of Xiong [9], only registered users can authenticate each other and build a shared key, besides, this shared key is only known by the two registered users and the network manager could not know this shared key. According to the public information transmitted between the two users, an adversary is unable to learn this shared key. The scheme of Jing et al. is a scheme equipped with elliptic curve cryptographic primitives. Their scheme achieves anonymity regardless of network infrastructure. Their scheme enables the server to provide various services for a client more than once with a negligible computational cost [10]. Idrissi proposed a security scheme for mobile agent based on two techniques: anonymous authentication and intrusion detection [11]. In the work of Xiong et al. [12], the anonymity is enabled, however the gateway has to store a lot of the identity and key pairs.

In some schemes, the gateway assigns a random number, and a unique key based on this number to the clients. This number is used as an indicator of the key, the user encrypts his identity with this key. Many other schemes use this way to protect the identity of the users, for example, the scheme in the works of [13,14,15,16,17,18]. Biometrics are used in the scheme of Wu et al. [19], Odelu et al. [20], Wang et al. [21] and Islam et al. [22]. Human beings’ biometrics are extracted as random strings by using the fuzzy extractor.

The partial public key method is a popular method that has been used. He et al. proposed an efficient identity-based privacy-preserving authentication scheme for vehicular ad hoc networks [23], batch verification is used in this study. The concept of partial public key is also used in the scheme of Islam et al. [24]. In their scheme, a user register at the server several times, in order to get more than one authentication keys, then the user can use different keys for authentication to achieve anonymity. The scheme of Porambage et al. [25] also used the partial public key concept. Tsai et al. proposed a scheme for distributed mobile cloud computing services [26], the security strength of their scheme is based on bilinear pairing and dynamic nonce generation. There are other schemes that based on the elliptic curve security [27,28,29].

## 3. The Proposed Scheme

### 3.1. Structure of the Scheme

**Figure 1:**
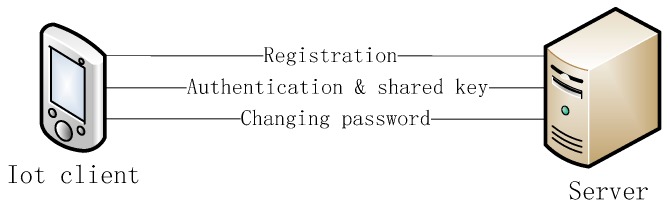
The structure of the proposed scheme.

There are two types of entities in the scheme: remote clients and the server, which is shown in Figure 1.

1. A client is the one who wants to access the services provided by the server. A client first registers at the server, after the registration, he can conduct a mutual authentication with the server, after authentication, the two can build a shared key, the client can access to the server’s service using this key.
2. A server is the one that provides different kinds of services to the client. A server is also responsible for the registration and password modification for the client. Before the server provides a service to a client, the server has to make sure if the client is a registered one or not.

The proposed scheme is a mutual authentication scheme between the client and the server. The scheme consists of three phases: registration phase of the client, mutual authentication and key establishment phase and the phase for a client to change his password.

### 3.2. System Initialization

In the beginning, the server S generates and publicizes the parameters of an elliptic curve, which is {p,a,b,P,n,h}. After that, S generates its private key $X_{GWN}$, and keeps it as a secret. The symbols that will be used in this study are summarized in Table 1.

### 3.3. Registration Phase

All the clients have to register at the server, a client $C_{i}$ with identity $ID_{i}$ generates a registration request message, and sends this request to the server S.

1. Client $C_{i}$ chooses a random number $r_{i}$.
2. Client $C_{i}$ calculates a hash message $MP_{i}=h\left(r_{i}\left||ID_{i}|\right|PW_{i}\right)$.
3. Client $C_{i}$ sends $\left\{ID_{i},\;MP_{i}\right\}$ to the server.

When the server S receives the message, server S generates the keys for client $C_{i}$, after that, the server S sends these keys to the client $C_{i}$. Table 2 is a description of the process.

1. Server S calculates a hash message $d_{i}=h(ID_{i}||X_{GWN})$.
2. Server S calculates $f_{i}=d_{i}⊕MP_{i}$.
3. Server S chooses a random number $k_{i}$.
4. Server S calculates a hash message $e_{i}=h(k_{i}||X_{GWN})$.
5. Server S calculates $h_{i}=e_{i}⊕MP_{i}$.
6. Server S sends $\left\{f_{i},h_{i},k_{i}\right\}$ and other system parameters to the client $C_{i}$.

### 3.4. Authentication Phase

If a client $C_{i}$ with identity $ID_{i}$ wants to ask a service from the server S, first, the two have to authenticate each other and build a shared key. The client $C_{i}$ inserts the smart card into a card reader, inputs his identity $ID_{i}^{\prime }$ and password $PW_{i}^{\prime }$. The smart card (SC) prepares the following message and sends it to the server S.

1. The client $C_{i}$ inserts its smart card into a card reader, inputs his identity $ID_{i}^{\prime }$ and password $PW_{i}^{\prime }$.
2. SC computes: $MP_{i}^{\prime }=h\left(r_{i}\left||ID_{i}^{\prime }|\right|PW_{i}^{\prime }\right)$.
3. SC uses $MP_{i}^{\prime }$ to get $d_{i}=f_{i}⊕MP_{i}^{\prime }$ and $e_{i}=h_{i}⊕MP_{i}^{\prime }$.
4. SC gets the current timestamp $T_{1}$ and the random number $k_{i}$.
5. SC gets a random number $k_{1}$ ∈ [1, *n* − 1], and calculates $A_{1}=k_{1}\cdot P$.
6. SC gets the hash $M_{1}=h\left(A_{1}||ID_{i}^{\prime }||k_{i}||d_{i}||T_{1}\right)$.
7. SC computes $M_{2}=(ID_{i}^{\prime }||M_{1})⊕e_{i}$.
8. Finally, SC sends {$k_{i},A_{1},M_{2},T_{1}$} to the server S.

When the server S receives the incoming message, it first checks the correctness of the message, after the verification, the server will generate the shared key between himself and the client. Then the server prepares the message for sending back to the client.

1. Server S checks the freshness of the $T_{1}$, if $T_{1}$ is not fresh, server S abandons the incoming message, the scheme ends here.
2. Server S calculates the key $h(k_{i}||X_{GWN})$ based on $k_{i}$.
3. Server S uses the key $h(k_{i}||X_{GWN})$ to decrypt $M_{2}$ to get $ID_{i}^{\prime }||M_{1}^{\prime }$, $ID_{i}^{\prime }||M_{1}^{\prime }=h(k_{i}||X_{GWN})⊕M_{2}$.
4. Server S calculates $d_{i}^{\prime }=h(ID_{i}^{\prime }||X_{GWN})$ based on the identity $ID_{i}^{\prime }$.
5. Server S checks if $M_{1}^{\prime }=h(A_{1}||ID_{i}^{\prime }||k_{i}||d_{i}^{\prime }||T_{1})$, if they are equal, the server accepts the incoming message, otherwise, the scheme terminates here.
6. Server S gets a random number $k_{2}$ ∈ [1, *n* − 1], and calculates $B_{2}=k_{2}\cdot P$.
7. Server S calculates the shared key $SK=h(k_{2}\cdot A_{1}||T_{1})$.
8. Server S calculates a new random number $k_{inew}=h_{1}(SK||T_{1})$.
9. Server S calculates a hash message $e_{inew}=h(k_{inew}||X_{GWN})$.
10. Server S calculates $M_{3}=h\left(B_{2}||e_{inew}||k_{inew}||d_{i}^{\prime }||SK\right)$.
11. Server S computes $M_{4}=(e_{inew}||M_{3})⊕h(d_{i}^{\prime }||T_{1})$.
12. Server S sends $\left\{B_{2},M_{4}\right\}$ to the client $C_{i}$.

When client $C_{i}$ gets the message $\left\{B_{2},M_{4}\right\}$, $C_{i}$ will do the following steps to authenticate the incoming message, if the client verifies the message, he will build a shared key with the server.

1. Client $C_{i}$ computes the shared key as $SK^{\prime }=h(k_{1}\cdot B_{2}||T_{1})$.
2. Client $C_{i}$ decrypts $M_{4}$ to get $e_{inew}^{\prime }||M_{3}^{\prime }=M_{4}⊕h(d_{i}||T_{1})$.
3. Client $C_{i}$ computes the random number $k_{inew}^{\prime }=h_{1}(SK^{\prime }||T_{1})$.
4. Client $C_{i}$ checks if $M_{3}^{\prime }=h\left(B_{2}||e_{inew}^{\prime }||k_{inew}^{\prime }||d_{i}||SK^{\prime }\right)$, if they are equal, $C_{i}$ accepts the shared key $SK^{\prime }$, and now client $C_{i}$ and the server S can communicate using the shared key $SK=SK^{\prime }$, otherwise the scheme terminates here.
5. Client $C_{i}$ updates $h_{i}=e_{inew}^{\prime }⊕MP_{i}^{\prime }$ and $k_{i}=k_{inew}^{\prime }$.

Now the client $C_{i}$ and the server S have authenticated each other and built a shared key. The Table 3 below depicts the whole process.

### 3.5. Password Change Phase

When a client $C_{i}$ wants to change his password, he can send a request to the server S, this request is sent in public channel. Table 4 is a description of this process.

1. The client $C_{i}$ inserts his smart card into a card reader, inputs his identity and password $ID_{i}^{\prime }$ and $PW_{i}^{\prime }$.
2. SC computes: $MP_{i}^{\prime }=h\left(r_{i}||ID_{i}^{\prime }||PW_{i}^{\prime }\right)$.
3. SC uses $MP_{i}^{\prime }$ to get $d_{i}=f_{i}⊕MP_{i}^{\prime }$ and $e_{i}=h_{i}⊕MP_{i}^{\prime }$.
4. SC gets the current timestamp $T_{1}$ and the random number $k_{i}$.
5. SC gets the hash $M_{1}=h(ID_{i}^{\prime }\left||k_{i}|\right|d_{i}||T_{1})$.
6. SC computes $M_{2}=(ID_{i}^{\prime }||M_{1})⊕e_{i}$.
7. Finally, SC sends {$k_{i},M_{2},T_{1}$} to the server S.

When the server S receives the message, server S will verify if the message is from a legitimate client, after that, the server S sends a replay to the client $C_{i}$.

1. Server S checks the freshness of the $T_{1}$, if $T_{1}$ is not fresh, server S abandons the incoming message.
2. Server S calculates the key $h(k_{i}||X_{GWN})$ based on $k_{i}$.
3. Server S uses the key $h(k_{i}||X_{GWN})$ to decrypt $M_{2}$ to get $ID_{i}^{\prime }||M_{1}^{\prime }$, $ID_{i}^{\prime }||M_{1}^{\prime }=h(k_{i}||X_{GWN})⊕M_{2}$.
4. Server S calculates $d_{i}^{\prime }=h(ID_{i}^{\prime }||X_{GWN})$ based on the identity $ID_{i}^{\prime }$.
5. Server S checks if $M_{1}^{\prime }=h(ID_{i}^{\prime }\left||k_{i}|\right|d_{i}^{\prime }||T_{1})$, if they are equal, the server verifies the incoming message, otherwise, the scheme terminates here.
6. Server S calculates $M_{3}=h(ID_{i}^{\prime }||d_{i}^{\prime }||k_{i}||T_{1})$.
7. Server S sends $\left\{M_{3}\right\}$ to the client $C_{i}$.

When a client $C_{i}$ receives the replay message from the server S, the smart card checks the correctness of this message, if it is from the server S, then the smart card will allow the client $C_{i}$ to input his new password.

1. SC checks if $M_{3}=h(ID_{i}^{\prime }\left||d_{i}|\right|k_{i}||T_{1})$, if they are equal, then the client is allowed to change his password.
2. SC computes $d_{i}=f_{i}⊕MP_{i}^{\prime }$ using the stored $f_{i}$ and the old $MP_{i}^{\prime }$.
3. SC computes $e_{i}=h_{i}⊕MP_{i}^{\prime }$ using the stored $h_{i}$ and the old $MP_{i}^{\prime }$
4. Client $C_{i}$ inputs the new password $PW_{i}^{*}$.
5. SC updates $MP_{i}^{\prime }$ to be $MP_{i}^{*}=h\left(r_{i}\left||ID_{i}|\right|PW_{i}^{*}\right)$.
6. SC uses this new $MP_{i}^{*}$ to update the stored version of $f_{i}$ and $h_{i}$ to get $f_{i}^{\prime }=d_{i}⊕MP_{i}^{*}$, $h_{i}^{\prime }=e_{i}⊕MP_{i}^{*}$.

## 4. Security Analysis by AVISPA

Automated Validation of Internet Security Protocols and Applications (AVISPA) is “a push-button tool for the automated validation of Internet security-sensitive protocols and applications” [30]. To test security features of the scheme in this study, we write the scheme in a role-based language called High-Level Protocols Specification Language (HLPSL), which is used for describing protocols and specifying their intended security features. The HLPSL code is listed in Appendix A.

We run the security check by using the CL-based Model-Checker [31], and the checker of On- the-Fly Model-Checker (OFMC) [32,33]. The simulation result shown in Table 5 demonstrates that the proposed scheme is safe.

## 5. Security Analysis Using BAN Logic

We conducted a security analysis of the proposed scheme using Burrows-Abadi-Needham Logic (BAN logic) [34]. By using BAN logic, we can determine whether the exchanged information is trustworthy, secure against eavesdropping. For more information on the symbols and primary postulates of BAN logic, please refer to our previous work [35].

### 5.1. The Premise and Proof Goals

Suppose there are two entities in the system: client $C_{i}$ and the server S. Before we start the proof, we first translate the messages into an idealized form of BAN logic, the results are shown in Table 6.

The goals in BAN Logic are described below. These goals can ensure $C_{i}$ and S to agree on a shared key SK.

$$1.\;C_{i}\;|\equiv C_{i}\overset{\;SK\;}{↔}S\;2.\;S\;|\equiv S\overset{\;SK\;}{↔}C_{i}$$

### 5.2. Assumptions

We make some assumptions to help us to prove the protocol; assumptions are listed in Table 7. First, we show the proof of assumption A1 and A3.

1. According to the “#()-introduction” rule, client $C_{i}$ creates $T_{1}$
  (1) $$C_{i}\;|\equiv \#\left(T_{1}\right)$$
2. According to (1) and the “promotion #” rule:
  (2) $$C_{i}\;|\equiv \#\left(M_{4}\right)$$
3. According to (2) and the “promotion #” rule:
  (3) $$C_{i}\;|\equiv \#\left(B_{2},M_{4}\right)$$
4. According to (3) and the “elimination of multipart messages” rule:
  (4) $$C_{i}\;|\equiv \#\left(B_{2}\right)$$

In this part, we show the proof of assumption A2 and A4. By checking the timestamp $T_{1}$, the server S can judge if $T_{1}$ is fresh or not, if $T_{1}$ is not fresh, the server S will abandon the message and the scheme ends here. Thus, we only consider the situation that server S believes timestamp $T_{1}$ is fresh, which is $S|\;\equiv \#\left(T_{1}\right)$.

5. According to the “promotion #” rule:
  (5) $$S\;|\equiv \#\left(k_{i},A_{1},M_{2},T_{1}\right).$$
6. According to (5) and the “elimination of multipart messages” rule:
  (6) $$S\;|\equiv \#\left(A_{1}\right)$$

After registration, both server S and the client $C_{i}$ believe that they have a shared key $d_{i}$. Translating into BAN Logic, we get assumptions A6: $S\;|\equiv C_{i}\overset{\;d_{i}\;}{↔}S$ and $C_{i}\;|\equiv S\overset{\;d_{i}\;}{↔}C_{i}$. We can get assumptions A5: $C_{i}\;|\equiv S\overset{\;h(d_{i}||T_{1})\;}{↔}C_{i}$ based on $C_{i}\;|\equiv S\overset{\;d_{i}\;}{↔}C_{i}$. Assumption A7 says that client $C_{i}$ believes server S has complete control over the data $B_{2}$, assumption A8 says that server S believes client $C_{i}$ has complete control over the data $A_{1}$.

### 5.3. The Proof of the Proposed Scheme

In this section, we start the proof. According to the message $\left\{k_{i},A_{1},{\left\{A_{1},ID_{i},k_{i},T_{1}\right\}}_{d_{i}},T_{1}\right\}$, which the client $C_{i}$ sends to server S, we can get the followings:

7. According to the message $\left\{k_{i},A_{1},{\left\{A_{1},ID_{i},k_{i},T_{1}\right\}}_{d_{i}},T_{1}\right\}$:
  (7) $$S⨞\left\{k_{i},A_{1},{\left\{A_{1},ID_{i},k_{i},T_{1}\right\}}_{d_{i}},T_{1}\right\}$$
8. According to (7) and “ ‘,’-elimination” rule:
  (8) $$S⨞{\left\{A_{1},ID_{i},k_{i},T_{1}\right\}}_{d_{i}}$$
9. According to (8), A6 and “|∼ introduction” rule:
  (9) $$S\;\left|\equiv C_{i}\right|\sim \left\{A_{1},ID_{i},k_{i},T_{1}\right\}$$
10. According to (9) and “ ‘,’-elimination” rule:
  (10) $$S\;\left|\equiv C_{i}\;\right|\sim A_{1}$$
11. According to A4, (10), and “|∼elimination” rule:
  (11) $$S\;\left|\equiv C_{i}\right|\equiv A_{1}$$
12. According to A8, (11), and “jurisdiction or control” rule:
  (12) $$S\;|\equiv A_{1}$$
13. As $k_{2}$ is randomly created by S, according to “#()- introduction” rule:
  (13) $$S\;|\equiv \#\left(k_{2}\right)$$
14. According to (13), A2, A4, and “#()- promotion” rule:
  (14) S |≡#(SK)
15. According to (11), (14), and “$\overset{\;k\;}{↔}$ introduction” rule:
  (15) $$S\;|\equiv S\overset{\;SK\;}{↔}C_{i}$$

Now we have proved the second goal, we will begin to prove the first goal by analyzing the message server S sends to client $C_{i}$: $\left\{B_{2},{\left\{e_{inew},B_{2},k_{inew},d_{i},SK\right\}}_{h(d_{i}||T_{1})}\right\}$.

16. According to the message $\left\{B_{2},{\left\{e_{inew},B_{2},k_{inew},d_{i},SK\right\}}_{h(d_{i}||T_{1})}\right\}$:
  (16) $$C_{i}⨞\left\{B_{2},{\left\{e_{inew},B_{2},k_{inew},d_{i},SK\right\}}_{h(d_{i}||T_{1})}\right\}$$
17. According to (16) and “ ‘,’-elimination” rule:
  (17) $$C_{i}⨞{\left\{e_{inew},B_{2},k_{inew},d_{i},SK\right\}}_{h(d_{i}||T_{1})}$$
18. According to (17), A5 and “|∼ introduction” rule:
  (18) $$C_{i}\;\left|\equiv S\right|\sim \left\{e_{inew},B_{2},k_{inew},d_{i},SK\right\}$$
19. According to (18) and “ ‘,’-elimination” rule:
  (19) $$C_{i}\;\left|\equiv S\right|\sim B_{2}$$
20. According to A3, (19), and “|∼elimination” rule:
  (20) $$C_{i}\;\left|\equiv S\right|\equiv B_{2}$$
21. According to A7, (20), and “jurisdiction or control” rule:
  (21) $$C_{i}\;|\equiv B_{2}$$
22. As $k_{1}$ is randomly created by $C_{i}$, according to “#()- introduction” rule:
  (22) $$C_{i}\;|\equiv \#\left(k_{1}\right)$$
23. According to (22), A1, A3, and “#()- promotion” rule:
  (23) $$C_{i}\;|\equiv \#\left(SK\right)$$
24. According to (20), (23), and “$\overset{\;k\;}{↔}$ introduction” rule:
  (24) $$C_{i}\;|\equiv C_{i}\overset{\;SK\;}{↔}S$$

Now, we have proved the two goals of the scheme. We can say that the proposed scheme is secure under BAN logic.

## 6. Formal Security Analysis

Suppose $G_{1}$ is a cyclic additive group of prime order q, P is the generator of $G_{1}$, the Elliptic Curve Computational Diffie–Hellman (*ECCDH*) problem is thought to be a computational hardness. The security of the shared key of the proposed scheme is based on the computational hardness of the *ECCDH* problem.

**Definition** **1.** *ECCDH problem. For any* $a,b,c\in Z_{q}^{*}$ *, given an instance* <aP,bP> *, it is computationally intractable to compute* cP=abP *.*

**Theorem** **1.** *The proposed scheme achieves shared key security if and only if the ECCDH problem is unable to be solved in polynomial time.*

We define the shared key security as that an adversary is unable to get the shared key between the client $C_{i}$ and server S based on the messages transferred publicly between them.

**Proof.**   

(⇒) Suppose there is an efficient algorithm $O_{I}$ which could break the *ECCDH* problem in probabilistic polynomial time. The adversary is able to get the messages publicly sent between the client $C_{i}$ and the server S: $\left\{k_{i},A_{1},M_{2},T_{1}\right\}$, and $\left\{B_{2},M_{4}\right\}$. Suppose $a\cdot P=A_{1}=k_{1}\cdot P$ and $P=B_{2}=k_{2}\cdot P$, adversary $A_{I}$ is able to get the $cP=k_{1}\cdot k_{2}\cdot P$ by using efficient algorithm $O_{I}$, the adversary is able to break the security of the shared key and get the shared key $h(k_{1}\cdot k_{2}\cdot P\;||T_{1})$.

(⇐) Suppose there is an efficient algorithm $O_{II}$ which could get the shared key between client $C_{i}$ and server S, as the hash operation is secure, the adversary has to get the shared key by calculating $k_{1}\cdot k_{2}\cdot P$. This means given $A_{1}=k_{1}\cdot P$ and $B_{2}=k_{2}\cdot P$, an adversary $A_{II}$ is able to get $k_{1}\cdot k_{2}\cdot P$. For the *ECCDH* problem, suppose $a\cdot P=A_{1}=k_{1}\cdot P$ and $b\cdot P=B_{2}=k_{2}\cdot P$, the adversary is able to get $c\cdot P=a\cdot b\cdot P=k_{1}\cdot k_{2}\cdot P$. This apparently contradicts the hardness of the *ECCDH* problem. □

**Theorem** **2.** *The proposed scheme achieves perfect forward privacy if and only if the ECCDH problem is unable to solve in polynomial time.*

**Proof.**   

The proof of perfect forward privacy is similar to Theorem 1. Even if the private key of the client is leaked to the adversary. What the adversary get is the same public information $\left\{k_{i},A_{1},M_{2},T_{1}\right\}$ and $\left\{B_{2},M_{4}\right\}$. Thus it is unable to get the past session key, neither. □

## 7. Comparison

In this section, we compared our scheme with related works in computation cost, computation at the registration phase and the authentication phase. The schemes are implemented in C++, the running codes have been upload to a public repository in the github.com [36]. The MIRACL C/C++ Library is used in this study [37], the library can be accessed at github.com [38]. The experiment is conducted in Visual Studio C++ 2017 on a 64-bits Windows 7 operating system, 3.5 GHz processor, 8 GB memory. The hash function is SHA-256, the symmetric encryption/decryption function is AES in MR_PCFB1 form, the 256-bit long key for symmetric encryption/decryption function is generated by SHA-256 hash operation. The Koblitz curve secp256k1 which is recommended by NIST is used in this study [39]. The parameters of this curve are listed in Appendix B. The code is compiled in x86 form, this simulation does not take into account the transmission of the data.

### 7.1. Computational Performance Analysis

First, we compared the computation costs of these schemes in the form of operation per phase, *T_H_*, *T_MUL_*, *T_ADD_*, *T_E/D_* are used for the computation cost for SHA-256 operation, element multiplication operation of $G_{1}$, element addition operation of $G_{1}$, and AES symmetric encryption/decryption operation. The results are listed at Table 8. As shown in the table, we can find that in all conditions, the computation cost of the proposed scheme is the minimal, as *T_MUL_* > *T_H_* and *T_E/D_* > *T_H_*. Thus, the proposed scheme has an advantage in the computation cost and energy consumption compared to related works. To test the analysis of the computation cost, we also simulated the schemes in the aforementioned environment respectively.

**Figure 2:**
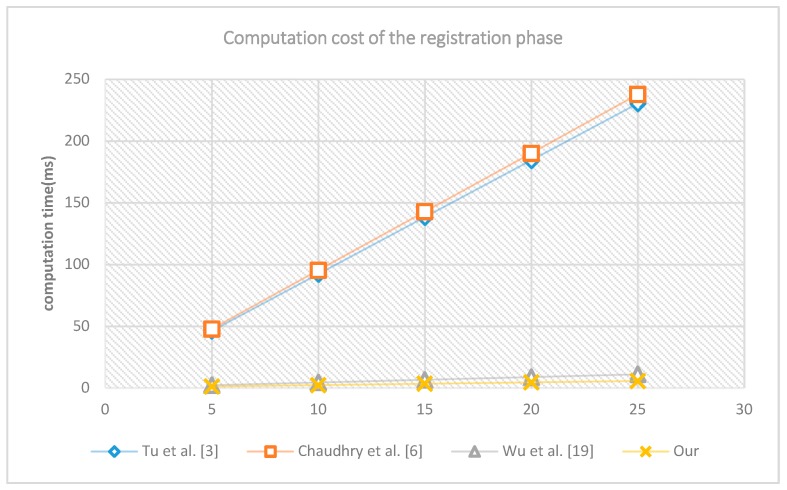
The computation cost of registration phase.

First, we run the registration phase of different schemes 5, 10, 15, 20 and 25 times separately. The computation times are shown in Figure 2. The horizontal axis represents the number of runs of the experiment, the vertical axis represents the time required for the experiment to run, and the unit is milliseconds. The computation cost of Wu et al. [19] and that of the proposed scheme are relatively smaller, while the scheme of Chaudhry et al. [6], and that of Tu et al. [3] cost more computation time. This is mainly because the proposed scheme and the scheme of Wu et al. [19] only need lightweight operations, SHA-256 hash operations and XOR operation, while for the scheme of Chaudhry et al. [6], and that of Tu et al. [3], symmetric encryption/decryption operations are required, these operations cost more computation time.

**Figure 3:**
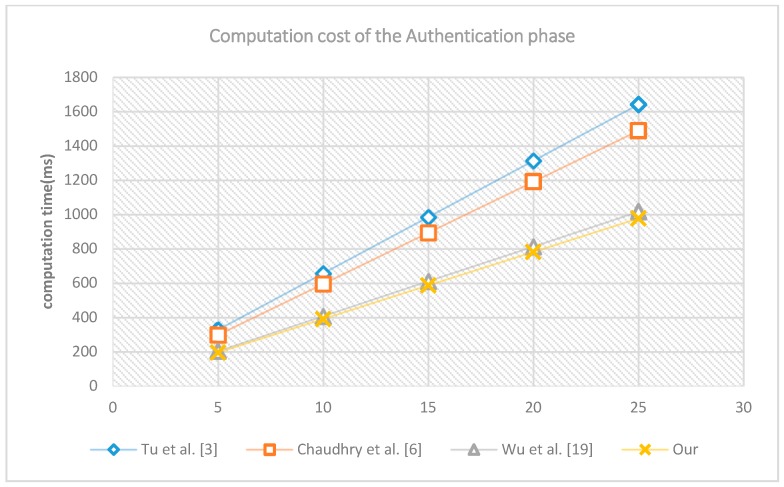
The computation cost of authentication phase.

Second, we run the authentication and key establishment phase of different schemes 5, 10, 15, 20 and 25 times separately. The computation costs are shown in Figure 3. The horizontal axis represents the number of running the experiment, the vertical axis stands for the number of milliseconds to accomplish the experiment. The computation cost of Wu et al. [19] and that of the proposed scheme are relatively smaller, while the scheme of Chaudhry et al. [6], and the scheme of Tu et al. [3] cost more computation time. The computation cost of the proposed scheme is the minimal.

**Figure 4:**
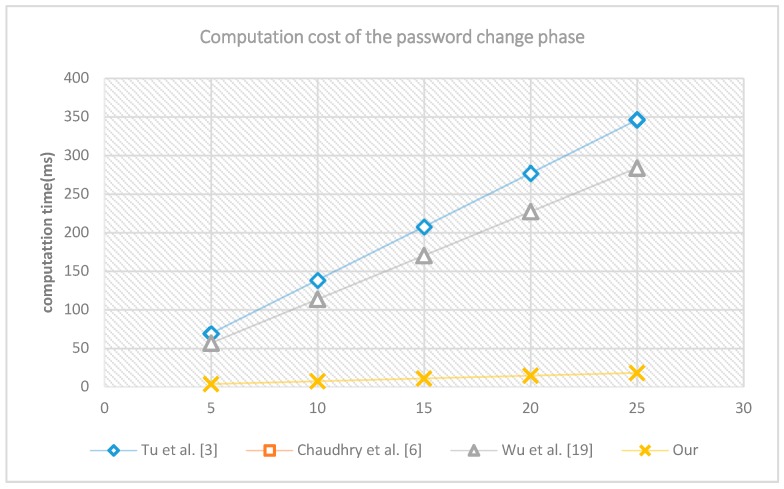
The computation cost of password change phase.

Third, we run the password change phase 5, 10, 15, 20 and 25 times separately. The computation costs are shown in Figure 4. In this figure, the horizontal axis indicates the number of times the experiment was run; the vertical axis indicates the number of milliseconds to accomplish the experiment. The computation cost of the proposed is the minimal, the computation cost of Wu et al. [19], and that of Tu et al. [3] are much higher, this is because in the proposed scheme only SHA-256 hash operations and XOR operation are needed, while in the scheme of Wu et al. [19], and in the scheme of Tu et al. [3], symmetric encryption/decryption, and elliptic curve operation are needed, these operations cost more computation time.

### 7.2. Communication Performance Analysis

In this part, we compared all the schemes in communication cost. We use the same criteria as that in the study of Jing et al. [8], the identity costs 2 bytes. The general hash operation in this study is SHA-256, the result of a hash operation is set to be 32 bytes. In this study, the random number is set to be 4 bytes, the timestamp is set to be 4 bytes. The element of the $G_{1}$ of the Koblitz curve secp256k1 is 64 bytes. The order |q| of $G_{1}$ is 32 bytes long.

At the registration phase, the client sends $\left\{ID_{i},MP_{i}\right\}$ to the server, $MP_{i}$ is a result of hash, it is 32 bytes long. The length of this message is 2 + 32 = 34 byte. The server sends $\left\{f_{i},h_{i},k_{i}\right\}$, $f_{i}$ is 32 byte long, $h_{i}$ is also 32 byte long. $k_{i}$ is 4 bytes a random number. The length of this message is 32 + 32 + 4 = 68 byte long. In the registration phase, the communication cost is 34 + 68 = 102 byte.

At the authentication phase, the client has to send $\left\{k_{i},A_{1},M_{2},T_{1}\right\}$ to the server, $k_{i}$ is a random number of be 4 bytes, $A_{1}$ is an element of $G_{1}$, it is 64 bytes long, $M_{2}=(ID_{i}^{\prime }||M_{1})⊕e_{i}$, $Id_{i}^{\prime }$ is an identity, it is 2 bytes long, $M_{1}$ is the result of an hash operation, it is 32 bytes long, the length of $M_{2}$ is 32 + 2 = 34 byte. $T_{1}$ is a 4 bytes long timestamp. The length of this message is 4 + 64 + 34 + 4 = 106. The server has to send $\left\{B_{2},M_{4}\right\}$ back to the client, $B_{2}$ is an element of $G_{1}$, it is 64 bytes long. $M_{4}=(e_{inew}||M_{3})⊕h(d_{i}^{\prime }||T_{1})$, $e_{inew}$ and $M_{3}$ are the results of hash, they are both 32 bytes long, the length of $M_{4}$ is 32 + 32 = 64 byte. The length of this message is 64 + 64 = 128 byte long. The communication cost of is 106 + 128 = 234 byte.

At the password change phase, the client has to send $\left\{k_{i},M_{2},T_{1}\right\}$ to the server, $k_{i}$ is a random number of be 4 bytes, $M_{2}=(ID_{i}^{\prime }||M_{1})⊕e_{i}$, $Id_{i}^{\prime }$ is an identity, it is 2 bytes long, $M_{1}$ is the result of an hash operation, it is 32 bytes long, the length of $M_{2}$ is 32 + 2 = 34 byte. $T_{1}$ is a 4 bytes long timestamp. The length of this message is 4 + 34 + 4 = 42. The server has to send $\left\{M_{3}\right\}$ back to the client, $M_{3}$ is the result of hash, it is 32 bytes long, the length of this message is 32 byte long. The communication cost of this phase is 42 + 32 = 74 byte.

The communication costs of other schemes are computed in the same way, note that, in the scheme of Tu et al. [3], to change a client’s password, the client and the server has to build a shared key in advance, thus, the communication cost of the password change phase is calculated as the communication cost of the authentication phase and the messages sent during the password change process. The scheme of Chaudhry et al. [6] does not have a password change phase; we did not calculate their scheme’s communication cost. The result is shown in Table 9.

## 8. Security Feature Analyses

In this section, we analyzed the security features of different schemes. At the end of this section, we concluded the results into a table.

### 8.1. Client Anonymity

Regarding client anonymity, in the proposed scheme, the identity of the user is encrypted by a shared key between the client and the server, the adversary is unable to find out the real identity of the client. In the scheme of Tu et al. [3], the identity of the user is transmitted transparently; the adversaries can get the identity easily. In the scheme of Chaudhry et al. [6] and Wu et al. [19], the identity is encrypted, too.

### 8.2. Perfect Forward Privacy

Perfect forward privacy means that even when an adversary gets the private key of the client or the server, it is unable to recover the past session key based on this private key and the publicly transmitted messages. As we have proved in Section 5, the proposed scheme gains perfect forward privacy.

Meanwhile, the scheme of Chaudhry et al. [6] cannot ensure perfect forward privacy, if the adversary gets the private key msk and the session related messages $DID_{ua},\;EID_{ua},\;Q_{ua}$ and $T_{sb},\;H_{sb}$. The adversary is able to compute the past session key in the following manner:

$$M_{ua}^{\prime }=msk\cdot Q_{ua}$$

$$EID_{ua}=M_{ua}^{\prime }⊕DID_{ua}$$

$$TID_{ua}^{\prime }=H_{1}\left(msk⊕ID_{ua}\right)\cdot P$$

$$Q_{sb}^{\prime }=T_{sb}⊕M_{ua}^{\prime }$$

$$SK=H_{5}\left(Q_{ua}⊕TID_{ua}^{\prime }⊕M_{ua}^{\prime }⊕TID_{ua}^{\prime }\right)$$

### 8.3. Reply Attack

In the proposed scheme, there is a timestamp $T_{1}$ in the message $\left\{k_{i},A_{1},M_{2},T_{1}\right\}$, and the timestamp $T_{1}$ is also concealed in the hash message $M_{1}=h\left(A_{1}||D_{i}^{\prime }||k_{i}||d_{i}||T_{1}\right)$. If an adversary sends a former message to the server, the server will abandon this message after checking the timestamp. However, if the adversary replaces the timestamp $T_{1}$ with a new one, the server can still find it out by checking the hash message $M_{1}=h\left(A_{1}||D_{i}^{\prime }||k_{i}||d_{i}||T_{1}\right)$. Thus, an adversary is unable to launch a replay attack. For the scheme of Chaudhry et al. [6], if an adversary sends a former message to the server, the server is unable to judge if the message is a previous one or not, therefore, their scheme is subjected to replay attack.

### 8.4. Offline Dictionary Attack

In the proposed scheme, if the adversary gets the message in the smartcard $\left\{f_{i},h_{i},k_{i},r_{i}\right\}$. The adversary could conduct an offline dictionary attack in the following steps:

1. The adversary insert the smart card into a card reader, inputs a random identity and password pair $ID_{i}^{\prime }$ and $PW_{i}^{\prime }$.
2. SC computes: $MP_{i}^{\prime }=h\left(r_{i}||ID_{i}^{\prime }||PW_{i}^{\prime }\right)$.
3. SC uses $MP_{i}^{\prime }$ to get $d_{i}=f_{i}⊕MP_{i}^{\prime }$ and $e_{i}=h_{i}⊕MP_{i}^{\prime }$.
4. SC gets the current timestamp $T_{1}$, and gets $k_{i}$.
5. SC gets a random number $k_{1}$ ∈ [1, *n* − 1], and calculates $A_{1}=k_{1}\cdot P$.
6. SC gets the hash $M_{1}=h\left(A_{1}||ID_{i}^{\prime }||k_{i}||d_{i}||T_{1}\right)$.
7. SC computes $M_{2}=(ID_{i}^{\prime }||M_{1})⊕e_{i}$.
8. Finally, SC sends {$k_{i},A_{1},M_{2},T_{1}$} to the server S.
9. If the server sends back a replay message, the identity and password pair is correct, otherwise, go to step 1.

Now, $q_{send}$ is used as the number of times an adversary can send a message to the server S in a time period, the server will set a limit on $q_{send}$, if the $q_{send}\;$ exceeds this preset limit, The server will no longer process the incoming messages from this adversary, the adversary cannot continuing the dictionary attack in this time period. The $\left|D_{id}\right|$, $\left|D_{pass}\right|$ are used as the dictionary size of the identity and the password. Thus the probability $p_{adv}$ that adversary correctly guesses the identity and password pair correctly is:

$$p_{adv}=\frac{q_{send}}{\left|D_{id}\right|*\left|D_{pass}\right|}$$

Set $\left|D_{id}\right|$, $\left|D_{pass}\right|$ to be large enough, the $p_{adv}$ will be a small value, the aforementioned analysis is based on the authentication phase, the attack on the password changing phase is the same.

Meanwhile, in the scheme of Chaudhry et al. [6], the adversary could conduct an offline dictionary attack in the following steps:

1. The adversary inserts the smart card into a card reader, inputs a random identity and password pair $ID_{i}^{\prime }$ and $PW_{i}^{\prime }$.
2. The adversary waits for the computation of the smart card.
3. If the smart card sends out a message, the identity and password pair is correct, otherwise, goes to step 1.

As there is not a limit, the adversary can try as many times as he wants, thus the adversary will finally get the correct identity and password pair. This also means our scheme can withstand the smart card lost attack, when the smart card is lost, the adversary cannot launch an offline dictionary attack to get the private key of the client.

### 8.5. Impersonation Attack

In the scheme of Tu et al. [3], an adversary can impersonate the server. Given the message a user sends to the server, {username,V,W}, an adversary can forge the following message, the user is unable to find out if this message is coming from an adversary or the server:

$$\mathrm{Generate}\;\mathrm{random}\;\mathrm{numnber}\;c,r\in Z_{n}\;C=c\cdot P,K=c\cdot V$$

$$SK=h_{1}\left(K\left||r|\right|username\right)$$

$$Auth_{s}=h_{2}(K||W||r||SK)$$

However, in the proposed scheme, if an adversary wants to impersonate the server, it has to get $d_{i}^{\prime }=h(ID_{i}^{\prime }||X_{GWN})$, the probablity that an adversary correctly guesses $d_{i}^{\prime }$ is $p_{d_{i}}=1/\left(\left|D_{id}\right|*\left|D_{X_{GWN}}\right|\right)$, where $\left|D_{X_{GWN}}\right|$ means the dictionary size of the server’s private key.

### 8.6. Secret Information Leakage Problem

In the scheme of Tu et al. [3], if an adversary accidentally get the session ephemeral information b. The adversary is able to get the secret information h(username||s)·P in the following manner:

$$h(username||s)\cdot P=b^{-1}\cdot V^{\prime }$$

With this secret information, the adversary can impersonate a legitimate client. However, in the proposed scheme, even the session ephemeral information is leaked, the adversary is unable to get the client’s secret information.

Finally, we get Table 10, we find that the proposed scheme has more security features than the schemes in the related works.

## 9. Conclusions

In this study, an authentication and key establishment scheme between remote clients and a server is proposed. The proposed scheme has been verified by AVISPA and BAN Logic, the verification results show that the proposed scheme can withstand various attacks. The proposed scheme has been simulated in C++, by comparison, it shows clearly that the proposed scheme is more efficient compared to the related works regarding the computation cost and the communication cost. Besides, the proposed has more security features compared to the related works. Our work is part of the LifeWear project, in which we focus on the safety of data transmission and identity privacy problem.

## Figures and Tables

**Table 1:** Symbols used in this study.

Symbols	Meaning
*S*	The server
$C_{i}$	The $i_{th}$ client
$ID_{i}$	The $i_{th}$ client’s identity
||	String connector, connecting two strings
⊕	XOR operation
*P*	The generator of ECC
$T_{1}$	Timestamp
*h*	The SHA-256 hash function
$h_{1}$	A hash a string to a random number function

**Table 2:** Registration phase.

Client	Server
$ID_{i},PW_{i}$	master key $X_{GWN}$
random number $r_{i}$	
$MP_{i}=h\left(r_{i}\left||ID_{i}|\right|PW_{i}\right)$	
$\left\{ID_{i},MP_{i}\right\}$ 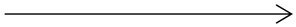	$d_{i}=h(ID_{i}||X_{GWN})$ $f_{i}=d_{i}⊕MP_{i}$ random number $k_{i}$
	$e_{i}=h(k_{i}||X_{GWN})$
	$h_{i}=e_{i}⊕MP_{i}$
Stores $\left\{f_{i},h_{i},k_{i}\right\}$	$\left\{f_{i},h_{i},k_{i}\right\}$ 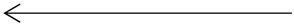

**Table 3:** Authentication phase.

Client	Server
$ID_{i},PW_{i}$	**Master Key** $X_{GWN}$
User: inserts SC into the terminal	
User: input $ID_{i}^{\prime }$ and $PW_{i}^{\prime }$	
SC: $MP_{i}^{\prime }=h\left(r_{i}\left|\left|ID_{i}^{\prime }\right|\right|PW_{i}^{\prime }\right)$	
SC: $d_{i}=f_{i}⊕MP_{i}^{\prime }$	
SC: $e_{i}=h_{i}⊕MP_{i}^{\prime }$	
SC: gets timestamp $T_{1}$, $k_{i}$	
Random number $k_{1}$, $A_{1}=k_{1}\cdot P$	
SC: gets $M_{1}=h\left(A_{1}\left|\left|ID_{i}^{\prime }\left|\left|k_{i}\right|\right|d_{i}\right|\right|T_{1}\right)$	
SC: $M_{2}=(ID_{i}^{\prime }||M_{1})⊕e_{i}$	
$\left\{k_{i},A_{1},M_{2},T_{1}\right\}$ 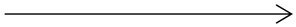	Checks the freshness of $T_{1}$ $ID_{i}^{\prime }||M_{1}^{\prime }=h(k_{i}||X_{GWN})⊕M_{2}$
	$d_{i}^{\prime }=h(ID_{i}^{\prime }||X_{GWN})$
	Check if $M_{1}^{\prime }=h\left(A_{1}\left|\left|ID_{i}^{\prime }\left|\left|k_{i}\right|\right|d_{i}^{\prime }\right|\right|T_{1}\right)$
	Random number $k_{2}$, $B_{2}=k_{2}\cdot P$
	$SK=h(k_{2}\cdot A_{1}||T_{1})$
	$k_{inew}=h_{1}(SK||T_{1})$
	$e_{inew}=h(k_{inew}||X_{GWN})$
	$M_{3}=h\left(B_{2}\left|\left|e_{inew}\right|\right|k_{inew}\left|\left|d_{i}^{\prime }\right|\right|SK\right)$
	$M_{4}=(e_{inew}||M_{3})⊕h(d_{i}^{\prime }||T_{1})$
$SK^{\prime }=h(k_{1}\cdot B_{2}||T_{1})$ $e_{inew}^{\prime }||M_{3}^{\prime }=M_{4}⊕h(d_{i}||T_{1})$	$\left\{B_{2},M_{4}\right\}$ 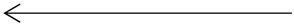
$k_{inew}^{\prime }=h_{1}(SK^{\prime }||T_{1})$	
Check if $M_{3}^{\prime }=h\left(B_{2}\left|\left|e_{inew}^{\prime }\right|\right|k_{inew}^{\prime }\left|\left|d_{i}\right|\right|SK^{\prime }\right)$	
$h_{i}=e_{inew}^{\prime }⊕MP_{i}^{\prime }$, $k_{i}=k_{inew}^{\prime }$	
Agree on the key $SK=SK^{\prime }$	

**Table 4:** Password change phase.

Client	Server
$ID_{i},PW_{i}$	**Master Key** $X_{GWN}$
User: inserts SC into the terminal	
User: input $ID_{i}^{\prime }$ and $PW_{i}^{\prime }$	
SC: $MP_{i}^{\prime }=h\left(r_{i}\left|\left|ID_{i}^{\prime }\right|\right|PW_{i}^{\prime }\right)$	
SC: $d_{i}=f_{i}⊕MP_{i}^{\prime }$	
SC: $e_{i}=h_{i}⊕MP_{i}^{\prime }$	
SC: gets timestamp $T_{1}$, $k_{i}$	
SC: gets $M_{1}=h(ID_{i}^{\prime }\left|\left|k_{i}\right|\right|d_{i}||T_{1})$	
SC: $M_{2}=(ID_{i}^{\prime }||M_{1})⊕e_{i}$	
$\left\{k_{i},M_{2},T_{1}\right\}$ 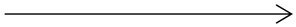	Check the freshness of $T_{1}$ $ID_{i}^{\prime }||M_{1}^{\prime }=h(k_{i}||X_{GWN})⊕M_{2}$
	$d_{i}^{\prime }=h(ID_{i}^{\prime }||X_{GWN})$
	Check if $M_{1}^{\prime }=h(ID_{i}^{\prime }\left|\left|k_{i}\right|\right|d_{i}^{\prime }||T_{1})$
	$M_{3}=h(ID_{i}^{\prime }||d_{i}^{\prime }\left|\left|k_{i}\right|\right|T_{1})$.
Check if $M_{3}=h(ID_{i}^{\prime }\left|\left|d_{i}\right|\right|k_{i}||T_{1})$ $d_{i}=f_{i}⊕MP_{i}^{\prime }$	$\left\{M_{3}\right\}$ 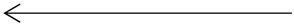
$e_{i}=h_{i}⊕MP_{i}^{\prime }$	
$MP_{i}^{*}=h\left(r_{i}\left|\left|ID_{i}\right|\right|PW_{i}^{*}\right)$	
$f_{i}^{\prime }=d_{i}⊕MP_{i}^{*}$	
$h_{i}^{\prime }=e_{i}⊕MP_{i}^{*}$	

**Table 5:** Simulation results of AVISPA.

CL-AtSe Back-End	OFMC
SUMMARY	% OFMC
SAFE	% Version of 2006/02/13
DETAILS	SUMMARY
BOUNDED_NUMBER_OF_SESSIONS	SAFE
TYPED_MODEL	DETAILS
PROTOCOL	BOUNDED_NUMBER_OF_SESSIONS
/home/iotdev/avispa/avispa-1.1/testsuite/results/light.if	PROTOCOL
	/home/iotdev/avispa/avispa-1.1/testsuite/results/light.if
GOAL	GOAL
As Specified	as_specified
	BACKEND
BACKEND	OFMC
CL-AtSe	COMMENTS
	STATISTICS
STATISTICS	parseTime: 0.00s
	searchTime: 0.01s
Analysed: 1 states	visitedNodes: 4 nodes
Reachable: 0 states	depth: 2 plies
Translation: 0.00 s	
Computation: 0.00 s	

**Table 6:** The idealized form of the messages.

Message	Flow	Idealized Form
1	$C_{i}\to S$	$\left\{k_{i},A_{1},{\left\{A_{1},ID_{i},k_{i},T_{1}\right\}}_{d_{i}},T_{1}\right\}$
2	$S\to C_{i}$	$\left\{B_{2},{\left\{e_{inew},B_{2},k_{inew},d_{i},SK\right\}}_{h(d_{i}||T_{1})}\right\}$

**Table 7:** Assumptions.

Number	Assumptions	Number	Assumptions
A1	$C_{i}\;|\equiv \#\left(T_{1}\right)$	A2	$S\;|\equiv \#\left(T_{1}\right)$
A3	$C_{i}\;|\equiv \#\left(B_{2}\right)$	A4	$S\;|\equiv \#\left(A_{1}\right)$
A5	$C_{i}\equiv S\overset{\;h(d_{i}||T_{1})\;}{↔}C_{i}$	A6	$S\equiv C_{i}\overset{\;d_{i}\;}{↔}S$
A7	$C_{i}\;|\equiv S⟾B_{2}$	A8	$S\;|\equiv C_{i}⟾A_{1}$

**Table 8:** Computation costs in the form of operation per phase.

Reference	Registration Phase	Authentication Phase	Password Change Phase
Tu et al. [3]	2*T_H_* + 1*T_MUL_*	10*T_H_* + 7*T_MUL_* + 1*T_ADD_*	6*T_H_* + 1*T_MUL_* + 4*T_E/D_*
Chaudhry et al. [6]	5*T_H_* + 1*T_MUL_*	14*T_H_* + 6*T_MUL_* + 1*T_ADD_*	---
Wu et al. [19]	4*T_H_*	12*T_H_* + 4*T_MUL_* + 4*T_E/D_*	9*T_H_* + 1*T_MUL_* + 2*T_E/D_*
Our scheme	3*T_H_*	14*T_H_* + 4*T_MUL_*	9*T_H_*

**Table 9:** Communication costs of different schemes.

Reference	Registration Phase	Authentication Phase	Password Change Phase
Tu et al. [3]	98 byte	230 byte	456 byte
Chaudhry et al. [6]	130 byte	226 byte	---
Wu et al. [19]	102 byte	238 byte	138 byte
Our scheme	102 byte	234 byte	74 byte

**Table 10:** Security features comparison.

Security Feature	Tu et al. [3]	Chaudhry et al. [6]	Wu et al. [19]	Our Scheme
Client anonymity	×	√	√	√
Client being tracked	×	√	√	√
Reply attack	×	×	×	√
Impersonation attack	×	√	√	√
Offline dictionary attack	√	×	√	√
Smart card lost attack	√	×	√	√
Changing password	√	×	√	√
Secret information leakage problem	×	√	√	√
Perfect forward privacy	√	×	√	√

## Appendix A

The role of the client.

role sender(Ui,Sj: agent,           Di,Ei: symmetric_key,           H  : hash_func,           P  : text,           SND_US,RCV_US : channel (dy))           SND_US,RCV_US : channel (dy)) played_by Ui def=  local State: nat, K1,T1,A1,IDi,Ki,M1,M2,SK,B2 ,Einew: text const user_server_sk,user_id:protocol_id  init State := 0 transition   1. State = 0  /\ RCV_US(start)=|>    State’:= 2  /\ Ki1’ := new()             /\ T1’:= new()             /\ A1’:= exp(P,K1’)             /\ M1’:= xor(Ei,(A1’.IDi))             /\ M2’:= H(A1’,IDi,Ki,Di,T1)             /\ SND_US(Ki.M1’.M2’.T1)   2. State = 2  /\ RCV_US( B2’.                        xor(                         (Einew’.                         H(H(exp(B2’,K1).T1).T1).                         H(B2’.                          Einew’.                          H(H(exp(B2’,K1).T1).T1).                          Di.                          H(exp(B2’,K1).T1))),                         H(Di,T1)                         )                       )=|>     State’:= 4  /\ SK’:= H(exp(B2’,K1).T1)             /\ Ei’:= Einew’             /\ Ki’:= H(H(exp(B2’,K1).T1).T1)              /\ secret(IDi,user_id,{Sj,Ui})             /\ witness(Ui,Sj,user_server_sk,SK’)             /\ request(Ui,Sj,user_server_sk,SK’) end role

The role of the server.

role server( Ui,Sj: agent,        Di,Ei :symmetric_key,        Xgwn :symmetric_key,        H  : hash_func,        P : text,        SND_US,RCV_US: channel(dy)) played_by Sj  def=  local State: nat,A1,T1,Ki,IDi,SK,K2,B2,Kinew,Einew,M3,M4: text const user_server_sk,user_id:protocol_id init State := 1 transition   1. State  = 1  RCV_US( Ki’.                       xor(H(Ki’.Xgwn),(A1’.IDi’)).                       H(A1’,IDi’,Ki’,Di’,T1’).                       T1’                       ) =|>    State’ := 3  /\ K2’ := new()             /\ B2’ := exp(P,K2’)             /\ SK’ := exp(A1’,K2’)             /\ Kinew’ := H(SK’,T1’)             /\ Einew’:= H(Kinew’,Xgwn)             /\ M3’ := H(B2’,Einew’,Kinew’,Di’,SK’)             /\ M4’ := xor((Einew’.Kinew’.M3’),H(Di’,T1’))             /\ SND_US( B2,M4’)              /\ secret(IDi,user_id,{Sj,Ui})             /\ witness(Sj,Ui, user_server_sk,SK’)             /\ request(Sj,Ui, user_server_sk,SK’) end role

The role of the session.

role session(Ui, Sj : agent,          Di,Ei, Xgwn : symmetric_key,          H : hash_func,          P : text) def=   local   SU,RU,SS,RS:channel(dy)  composition   user  (Ui,Sj, Di,Ei,    H,P, SU,RU) /\ server (Ui,Sj, Di,Ei,Xgwn, H,P, SS,RS) end role

The role of the environment.

role environment() def= const ui,sj : agent, di,xgwn,dii,ei: symmetric_key, user_server_sk,user_id:protocol_id, h : hash_func, p : text  intruder_knowledge={ui, sj, dii,eii,xgwni, h,p}  composition        session(ui,sj,  di,ei,xgwn, h,p)       /\ session( i,sj, dii,eii,xgwn, h,p)       /\ session(ui, i, di,ei,xgwni, h,p) end role

The role of the goals.

goal % Confidentiality (G12) secrecy_of user_server_sk,user_id  % Message authentication (G2) authentication_on user_server_sk end goal

## Appendix B

The parameters of the Koblitz curve secp256k1 by NIST are listed in this part. The curve is defined as $E:y^{2}=x^{3}+ax+b$ over $F_{p}$. The bit length of p is 256 bit.

*p* = FFFFFFFF FFFFFFFF FFFFFFFF FFFFFFFF FFFFFFFF FFFFFFFF FFFFFFFE FFFFFC2F
*a* = 0
*b* = 7
$G_{x}$ = 79BE667E F9DCBBAC 55A06295 CE870B07 029BFCDB 2DCE28D9 59F2815B
$G_{y}$ = 483ADA77 26A3C465 5DA4FBFC 0E1108A8 FD17B448 A6855419 9C47D08F
*n* = FFFFFFFF FFFFFFFF FFFFFFFF FFFFFFFE BAAEDCE6 AF48A03B BFD25E8C D0364141
*h* = 01
